# General Reference and Design S–N Curves Obtained for 1.2709 Tool Steel

**DOI:** 10.3390/ma16051823

**Published:** 2023-02-23

**Authors:** Michał Böhm, Adam Niesłony, Szymon Derda, Robert Owsiński, Miloslav Kepka, Ivana Zetkova, Miroslav Zetek, Šárka Houdková, Mariusz Prażmowski

**Affiliations:** 1 Department of Mechanics and Machine Design, Faculty of Mechanical Engineering, Opole University of Technology, Mikołajczyka 5, 45-271 Opole, Poland; 2 Regional Technological Institute, Faculty of Mechanical Engineering, University of West Bohemia, Univerzitní 8, 301 00 Plzeň, Czech Republic; 3 Research and Testing Institute in Plzeň, Tylova 1581/46, 301 00 Plzeň, Czech Republic

**Keywords:** fatigue of materials, S–N curves, 1.2709 steel, 3D-printed materials, SLM 3D printing

## Abstract

At present, due to advanced fatigue calculation models, it is becoming more crucial to find a reliable source for design S–N curves, especially in the case of new 3D-printed materials. Such obtained steel components are becoming very popular and are often used for important parts of dynamically loaded structures. One of the commonly used printing steels is EN 1.2709 tool steel, which has good strength properties and high abrasion resistance, and can be hardened. The research shows, however, that its fatigue strength may differ depending on the printing method, and may be characterized by a wide scatter of the fatigue life. This paper presents selected S–N curves for EN 1.2709 steel after printing with the selective laser melting method. The characteristics are compared, and conclusions are presented regarding the resistance of this material to fatigue loading, especially in the tension–compression state. A combined general mean reference and design fatigue curve is presented, which incorporates our own experimental results as well as those from the literature for the tension–compression loading state. The design curve may be implemented in the finite element method by engineers and scientists in order to calculate the fatigue life.

## 1. Introduction

The proper use of S–N curve data is one of the most important steps in the process of fatigue life assessment. The reliability of the results depends on the engineer’s experience, but mostly on the quality of the S–N data. Therefore, it is important to work on reliable data sets when implementing S–N curves in the fatigue life estimation process, usually together with the finite element method (FEM). The process is especially interesting when dealing with 3D-printed materials. EN 1.2709 tool steel can be found in the literature under different descriptions and names such as EOS MS1: US classification 18% Ni Maraging 300, European 1.2709 and German X3NiCoMoTi 18-9-5. It is used in the additive manufacturing process of 3D elements. EOS MS1 is delivered only as a powder. The material is praised for its good strength properties and high abrasion resistance as well as its potential to be hardened. Due to the fact that the material exists under different names, it is sometimes difficult to find the required experimental data in order to predict the fatigue life of designed components printed using it. Due to the 3D printing technology, an increasing number of mechanical elements are being designed and produced with the selective laser melting method. This method is regarded as the most promising in terms of additive manufacturing. The analysis performed within this paper consists of fatigue S–N data that were obtained for specimens produced with the use of the selective laser melting (SLM) method. The influence of different parameters was taken into account during the data analysis. Parameters such as different heat treatments and defect sizes and locations as well as the specimen smoothness and stress concentrators in the specimen shape and geometry were also described for the literature data. All data analyses were performed for the case of cyclic constant amplitude stress loading conditions. This served in the process of the comparison between the S–N curves taken from the literature and the obtained experimental curve for the tension–compression tests.

As we sorted through the literature, we noticed that papers about the fatigue life of EN 1.2709 tool steel are difficult to find, especially due to the fact that the steel can be found under different names. After noticing this, we observed that this topic is being pursued by some researchers, especially for 3D printing with the SLM technology. In this section, a number of related papers are presented, which take into account different parameters that influence the obtained experimental fatigue results; some of them also refer to the powder bed fusion (PBF) process or direct metal laser sintering (DMLS), which are equal to the SLM technology. Interesting papers that discuss the maraging steel fatigue properties for casted specimens are also mentioned. The authors mostly focus on papers that published an S–N curve. We can divide the papers into subcategories that present parameters such as:

- Different heat treatments;
- Defect size and location;
- Powder contamination;
- Specimen shape and build orientation;
- Loading type;
- Non-zero mean stress loading conditions.

As for different heat treatments, we found a large number of papers, such as the paper by Bouzakis et al. [1], where the authors additionally discussed the corrosion fatigue problem. Another group of authors led by Kostic et al. [2] analyzed the effect on heat-treated and untreated specimens under bending loading. Elangeswaran et al. [3] also focused on the heat treatment effects as well as the surface post-treatment processes that might increase the fatigue life. It can be concluded that heat treatment increases the fatigue life of specimens.

As for the defect size and location, we found the paper by Bai et al. [4], where the authors discussed the influence of hole-type defects inside the printed material. No specific S–N curve was presented, which could have been widely discussed or used in the analysis. It may be concluded that laser power has a positive effect on the surface quality, which serves in prolonging the fatigue life of specimens.

As for powder contamination, we found the paper by Gatto et al. [5], where the authors analyzed this influence on fatigue life in comparison with forged specimens. They stated that powder contamination has a great influence on the fatigue life of 3D-printed specimens in terms of its decrease. The presented experimental results were found for the case of non-zero mean stress under tension–compression.

As for the specimen shape, we found the papers by Branco et al., where the authors discussed the effect of notches on the fatigue life [6,7]. This effect of a stress concentrator in the specimen affects the fatigue life performance of SLM-printed materials. Dörfert et al. [8] discussed the fatigue of conventionally obtained and 3D-printed materials. The S–N curves showed that the conventionally obtained material had higher fatigue strength characteristics than the 3D-printed material. Fitzka et al. [9] analyzed the size effect in terms of the fatigue life for two different sizes of sheet specimens that were tested under ultrasonic frequencies obtained from a coil material. Guo et al. [10] presented a review in which they compared the fatigue results of 3D-printed specimens for notched and un-notched cases, as well as for cases using PBF printing technology. They presented an S–N curve for a variety of experimental results under tension–compression taken from the literature. Meneghetti et al. [11] presented a paper focused mostly on the effects of the build orientation of specimens and heat treatments, along with the fatigue results for the tension–compression loading state with the use of DMLS. Solberg et al. [12] discussed the effect of the directional build orientation under tension–compression for plate specimens. The comparison was performed for different build angles, and surfaces were as-built during the printing process or machined. The machined specimens had superior fatigue properties in comparison to the as-built ones. Tshabalala et al. [13] also performed a tension–compression test for cylindrical specimens with different build orientations. They observed that tool steels develop compressive mean stress during axial tests. It can be concluded that the shape, dimensions and distortion in terms of stress concentrators in the form of notches can cause a decrease in the fatigue life.

As for the loading type, we found the paper by Branco et al. [14], where the authors presented fatigue results under constant and variable amplitude loading. The same authors discussed the problem of multiaxial loading conditions concerning the studied material [15]. Crocollo et al. [16] presented experimental results for specimens under bending loading conditions. They focused on the sensitivity of the technology in regard to the build orientation. The paper by Damon et al. [17] presented tension–compression fatigue results under different temperatures and discussed the effect of the vertical and horizontal orientation of the printing procedure. Vilchez et al. [18] presented the fatigue results for maraging steel 300 in the tension–compression state under ultrasonic frequencies. The material was not printed but obtained from solid bars, but the obtained results showed the enormous possibilities in terms of the obtained fatigue life. It can be concluded that variable loads can cause a decrease in the fatigue life of specimens.

As for non-zero mean stress loading conditions, we found the paper by Chang [19] et al., where the authors presented multiple S–N curves for four different cycle asymmetry ratios, R. Their paper also strongly focused on the description of the defects that may influence the fatigue life of the components, as well as the temperature effects. Nevertheless, these tests were performed on a casted material, where the specimens were obtained from casted rods. Schuller et al. [20] presented fatigue S–N curves for two different R values under ultrasonic loading tests. It may be concluded that positive mean stresses influence the fatigue life of printed specimens by decreasing their lifetime.

Summarizing the literature review, it is found that the material has been tested by various scientists under different conditions. The shortcoming is the fact that it is difficult to formulate a “safe” design curve on the basis of individual literature results. It is important to note that the authors analyzed the fatigue results for 3D-printed specimens of this steel under different loading states inter alia for bending and tension–compression. The study presents never-before-published experimental fatigue results for 3D-printed specimens of 1.2709 tool steel under tension–compression on a fatigue test stand under the stress ratio R = −1. The most important part of this paper is the presentation of new experimental fatigue results as well as the presentation of a mean reference curve for the obtained experimental data and literature data as well as a reference design curve that was prepared according to the British Standard: BS 7608:1993 for the same data. There are currently no reference curves that represent a data set as large as the one presented within this paper. The design curve may be implemented in FEM by engineers and scientists in order to calculate the fatigue life in a reliable way, especially for simple objects that have been printed, e.g., plates with the dimensions reported by Van Vihn et al. [21].

## 2. Materials and Methods

**Figure 1:**
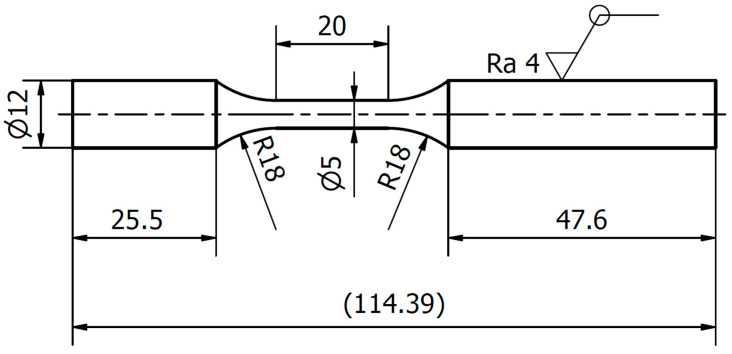
Geometry and dimensions of the specimens used in this experimental research for the tension–compression tests.

The material used in the experimental investigation was EN 1.2709 tool steel. The mechanical properties of this steel are presented in Table 1. The powder of this material was used in order to print specimens, whose geometry and dimensions are presented in Figure 1. The specimens were not subject to additional machining, but they were in the state after solution annealing.

**Figure 2:**
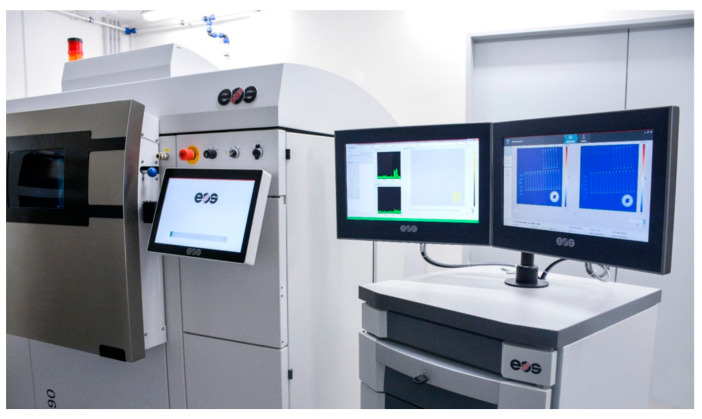
Selective laser sintering 3D printing machine EOS M290 used in the process of specimen preparation.

The machine used during the printing process was the EOS M290 with EOSTATE Exposure OT, MeltPool Monitoring systems and is presented in Figure 2. The default EOS parameter set was used for the printing of the samples in the Z direction (laser power of 258 W, scanning rate of 960 m/s, hatching of 0.11 mm, layer thickness of 40 micrometers, protective atmosphere of nitrogen gas). The samples were tested after solution annealing (temperature of 820 °C/1 h, slow cooling in the furnace).

**Figure 3:**
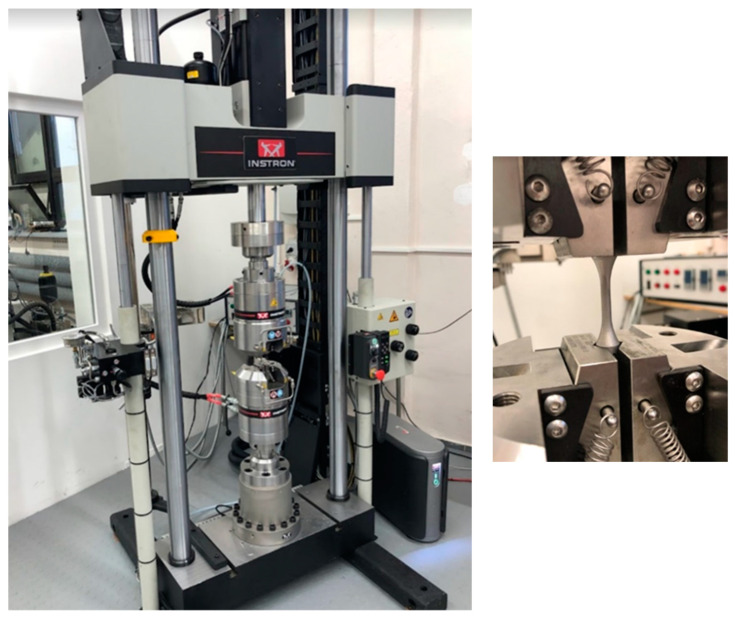
Fatigue test stand for tension–compression axial loading, Instron 8852, at Opole University of Technology.

**Figure 4:**
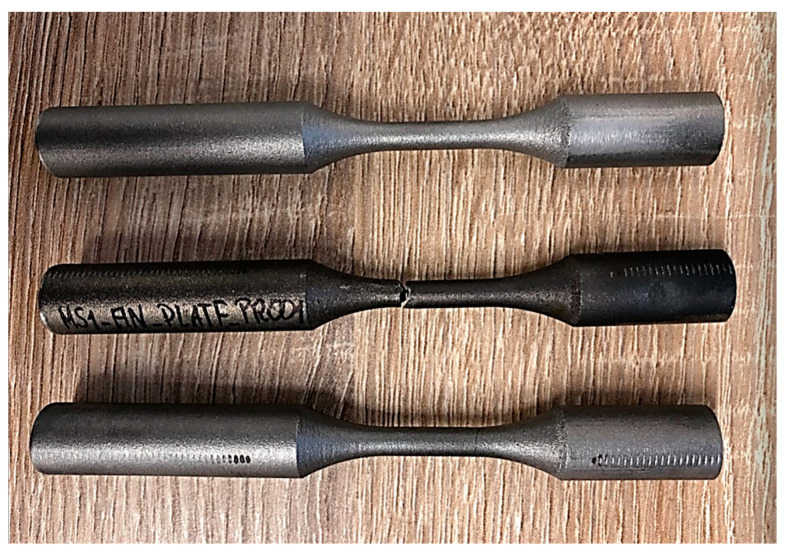
Photograph of test specimens before and after axial fatigue testing, OUTech Opole.

The specimens underwent fatigue tests under tension–compression performed on the Instron 8852 test stand presented in Figure 3. The tests were performed with a zero mean stress value. Therefore, no initial compression or tension was added. In Figure 4, a photograph of the specimens before and after the fatigue tests is shown.

The basic formula used for the description of the S–N curve is

(1) $$N=B{\left(\sigma_{a}\right)}^{-m},$$

where *N* is the number of cycles until failure, *B* is the S–N curve constant and *m* is the slope of the S–N curve.

An examination of the microstructure was also performed. The metallographic specimens were ground with abrasive papers or diamond pastes with decreasing gradation. Then, they were polished using an aqueous suspension of Al_2_O_3_ and etched using an etching reagent (10 mL HF, 30 mL HNO_3_, 50 mL H_2_O).

## 3. Results

**Figure 5:**
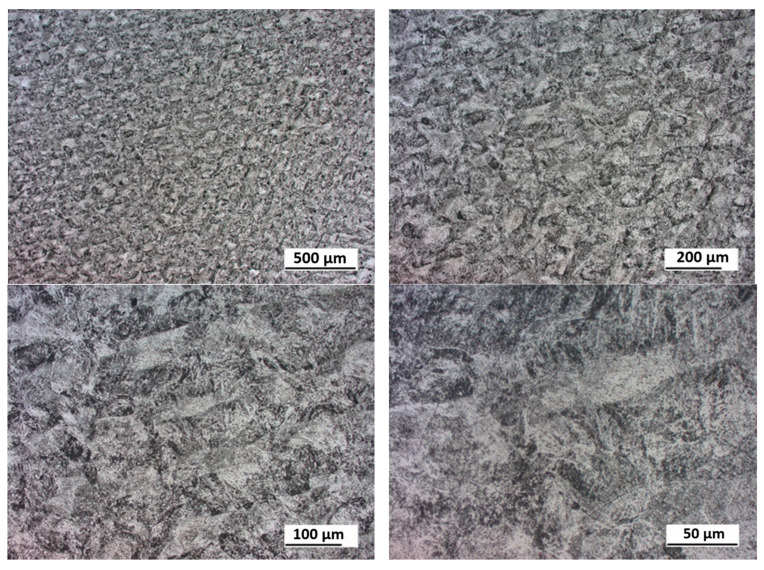
Microstructure of the 3D-printed specimens under different magnifications.

**Figure 6:**
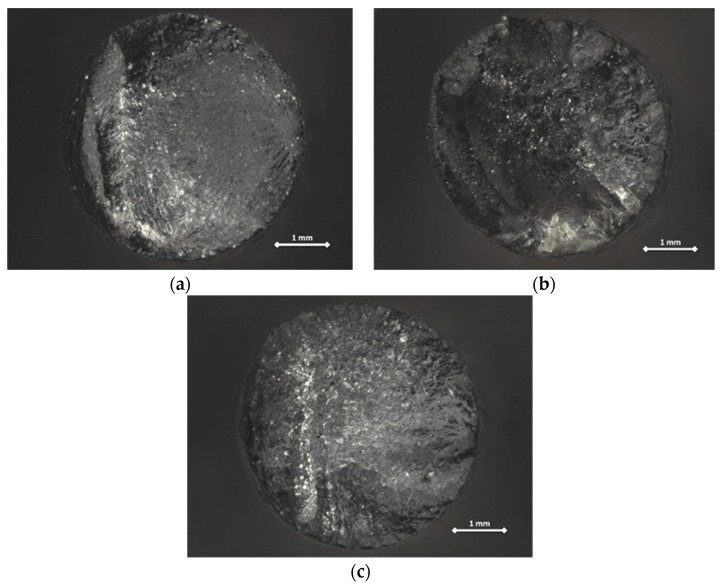
Fatigue fracture surfaces of three specimens under different loading amplitudes: (**a**) MS1_AN_PLATF_5_Pr004; (**b**) MS1_AN_PLATF_5_Pr012; (**c**) MS1_AN_PLATF_5_Pr015.

**Figure 7:**
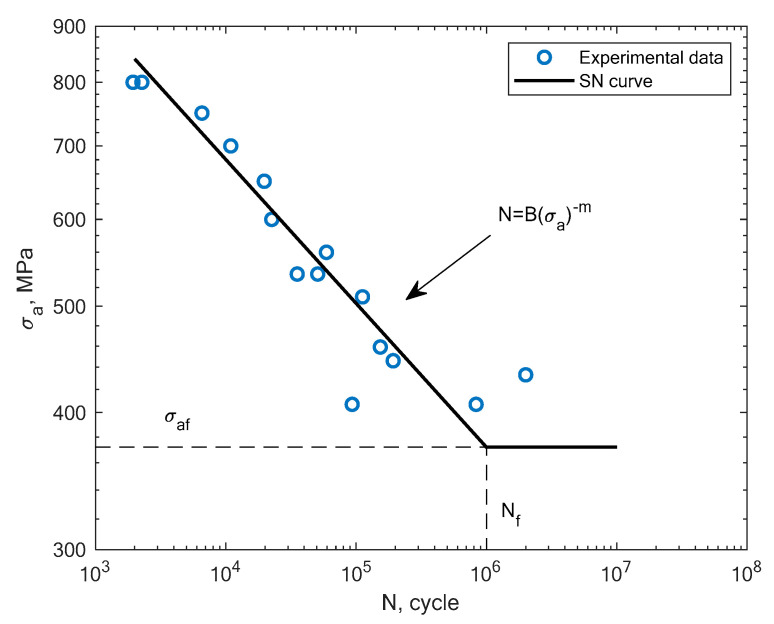
S–N curve for the experimental tension–compression fatigue test results of 1.2709 steel obtained by the authors under R = −1.

In this section, the obtained experimental results and S–N curves are presented, as well as the S–N curves taken from the literature in order to compare other states of loading conditions. The results of the experimental fatigue tests are presented in Table 2. The microstructure of the material is presented in Figure 5. The microstructure resembles a Widmanstätten austenite structure. The Widmanstätten structure is formed in the weld after remelting and cooling. This might be the case for this material as the SLM method works on the basis of this principle. In Figure 6, the fatigue fracture surfaces of three specimens under different loading amplitude values are presented. It can be noticed that the fractures obtained using the low-cycle (MS1_AN_PLATF_5_Pr012 and MS1_AN_PLATF_5_Pr015) and high-cycle regimes (MS1_AN_PLATF_5_Pr004) in the fatigue curve have an expected fracture area of propagation and initiation on the surface of the specimens, which is the expected case for the tension–compression loading state. In Figure 7, the authors present the S–N curves obtained for the tension–compression loading state under R = −1 for smooth cylindrical specimens.

**Figure 8:**
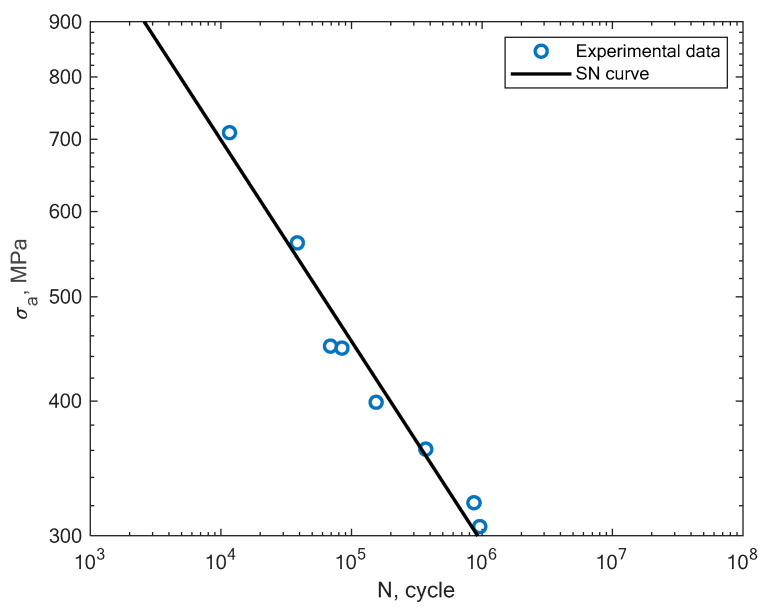
S–N curve for the experimental tension–compression fatigue test results (R = −1) reported by Branco et al. [14].

Beside the experimental results of the fatigue tests, this section also presents an analysis of different S–N curves obtained for additively manufactured specimens reported by other authors. A Wöhler diagram is used to describe the fatigue life. The diagram presents the fatigue life in terms of cycles until failure *N_f_* on the x axis. The constant stress amplitude *σ_a_* on the y axis represents the value at which the material is loaded during the constant tests. The literature sometimes presents the S–N diagrams in relation to the maximum stress *σ_max_*. In Figure 8, the experimental results of the tension–compression tests under R = −1 performed by Branco et al. [14] for specimens created with the use of the SLM technology are shown.

**Figure 9:**
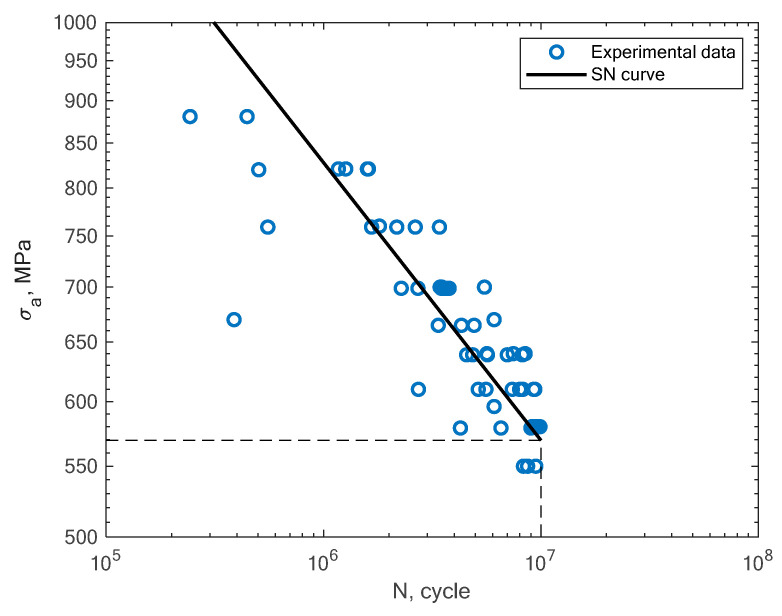
S–N curve for the experimental rotating bending fatigue test results reported by Croccolo et al. [16].

**Figure 10:**
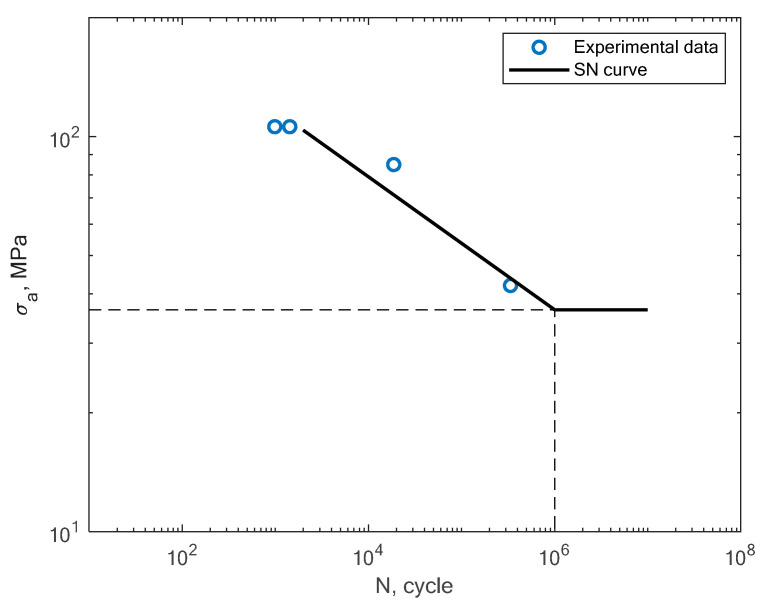
S–N curve for the experimental tension–compression fatigue test results reported by Cruces et al. [7].

In Figure 9, the experimental results obtained in the work of Croccolo et al. for specimens created with the direct metal laser sintering method and tested under a rotating bending load (R = −1) are shown [16]. The authors tested the sensitivity of the material in terms of the build direction. In Figure 10, the experimental results obtained by Cruces et al. for the tension–compression (R = −1) of smooth specimens with hollow cylinders are shown [7]. The authors analyzed smooth specimens with two differently sized circular stress concentrators. It can be noticed that their results apply to notched specimens with 0.4 and 1 mm notches.

**Figure 11:**
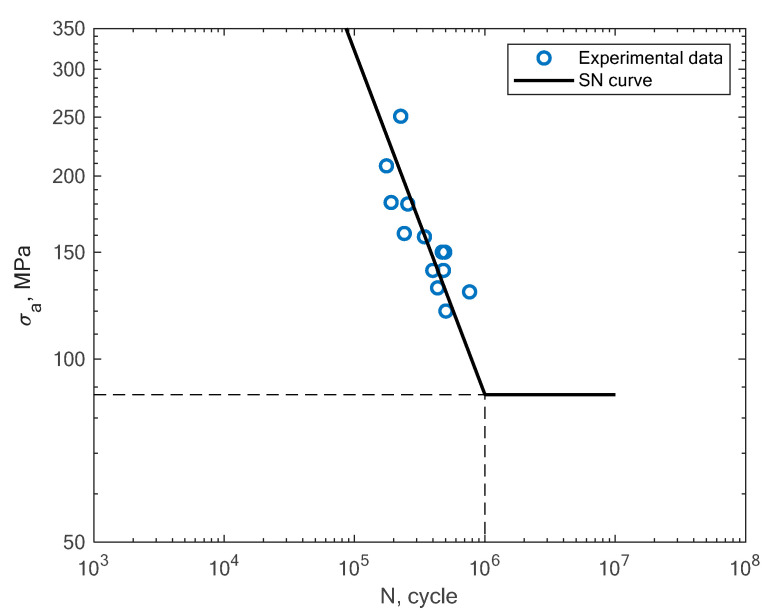
S–N curve for the experimental bending fatigue test results reported by Kostic et al. [2] for the case of no heat treatment and no machining.

**Figure 12:**
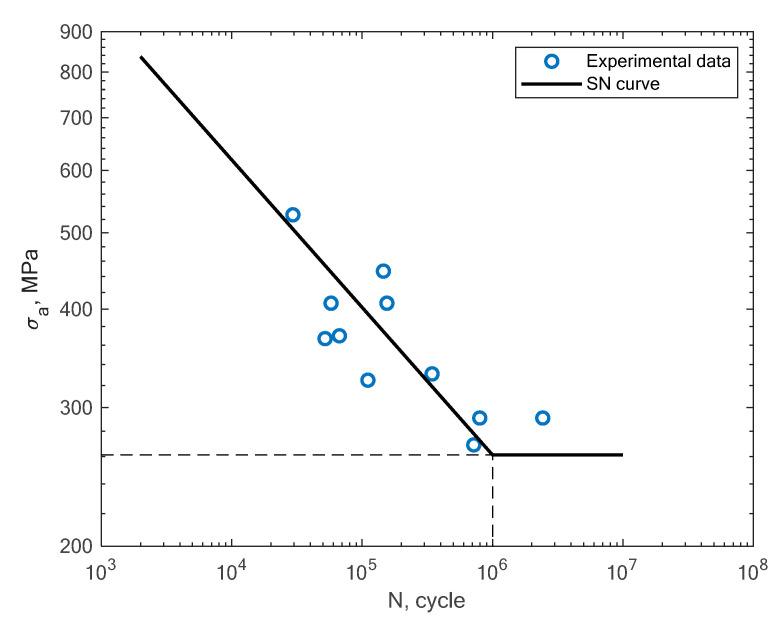
S–N curve for the experimental bending fatigue test results reported by Kostic et al. [2] for the case of heat treatment and no machining.

**Figure 13:**
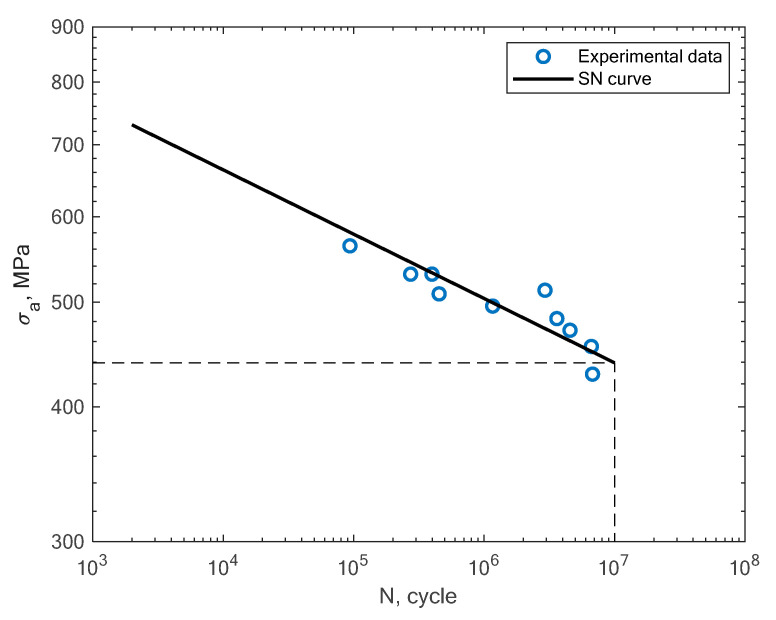
S–N curve for the experimental bending fatigue test results reported by Kostic et al. [2] for the case of no heat treatment and machining.

**Figure 14:**
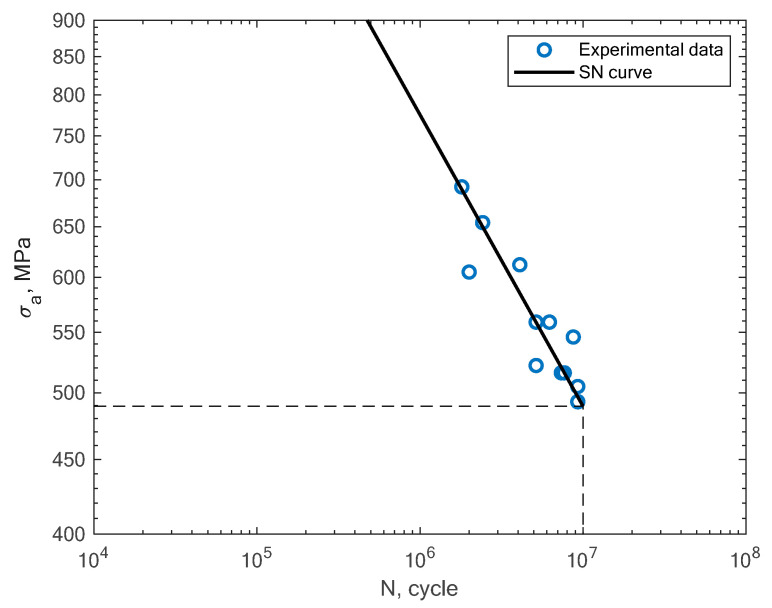
S–N curve for the experimental bending fatigue test results reported by Kostic et al. [2] for the case of heat treatment and machining.

**Figure 15:**
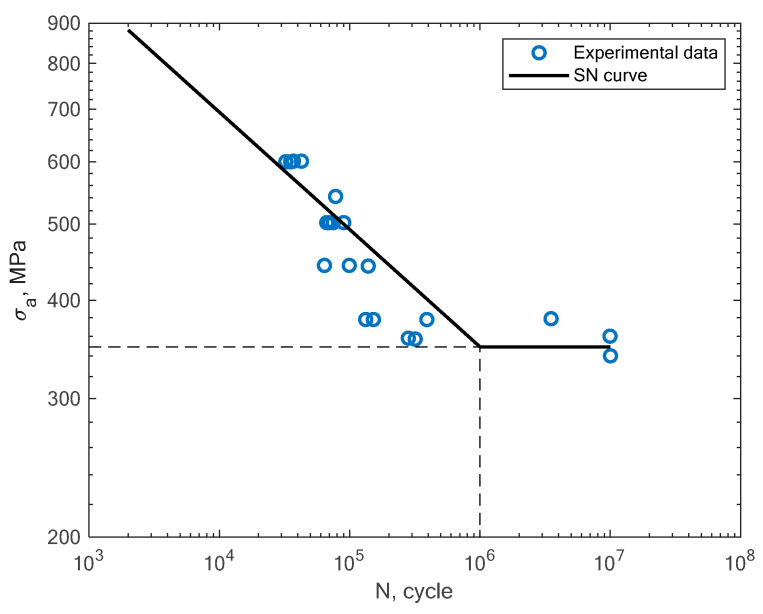
S–N curve for the experimental tension–compression (R = −1) fatigue test results reported by Damon et al. [17] under the conditions of room temperature and a horizontal direction of the printing orientation.

**Figure 16:**
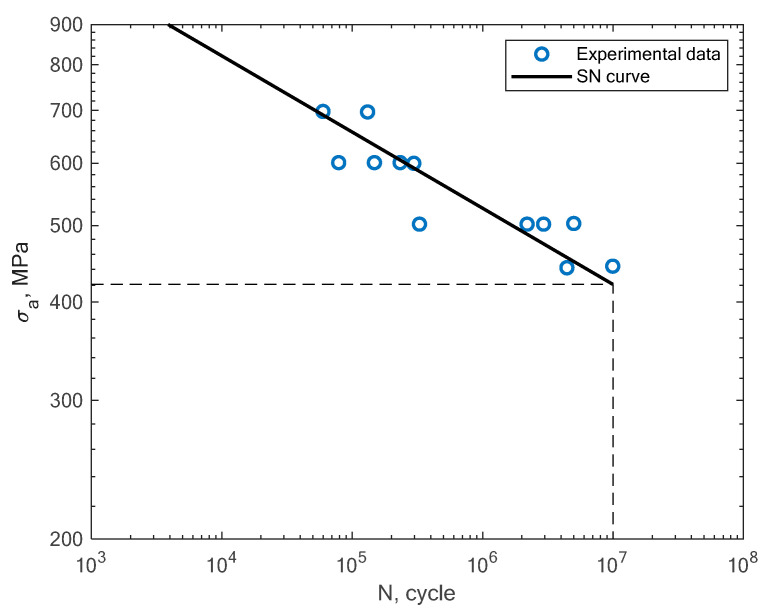
S–N curve for the experimental tension–compression (R = −1) fatigue test results reported by Damon et al. [17] under the conditions of room temperature and a vertical direction of the printing orientation.

**Figure 17:**
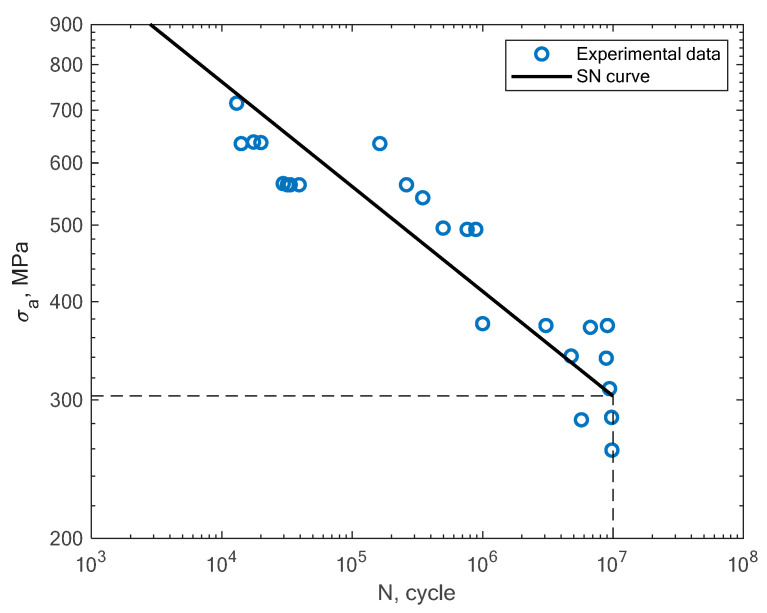
S–N curve for the experimental tension–compression (R = −1) fatigue test results reported by Damon et al. [17] under the conditions of a high temperature of 400 °C and a horizontal direction of the printing orientation.

**Figure 18:**
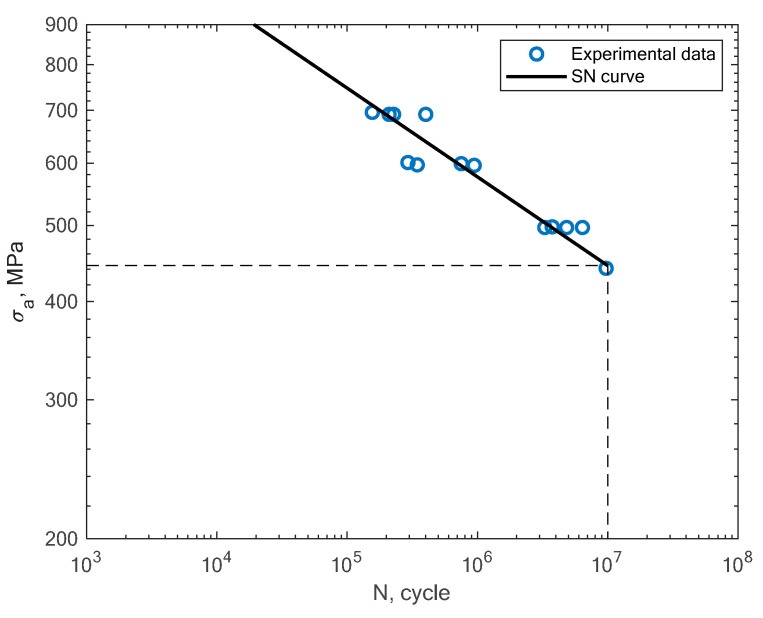
S–N curve for the experimental tension–compression (R = −1) fatigue test results reported by Damon et al. [17] under the conditions of a high temperature of 400 °C and a vertical direction of the printing orientation.

**Figure 19:**
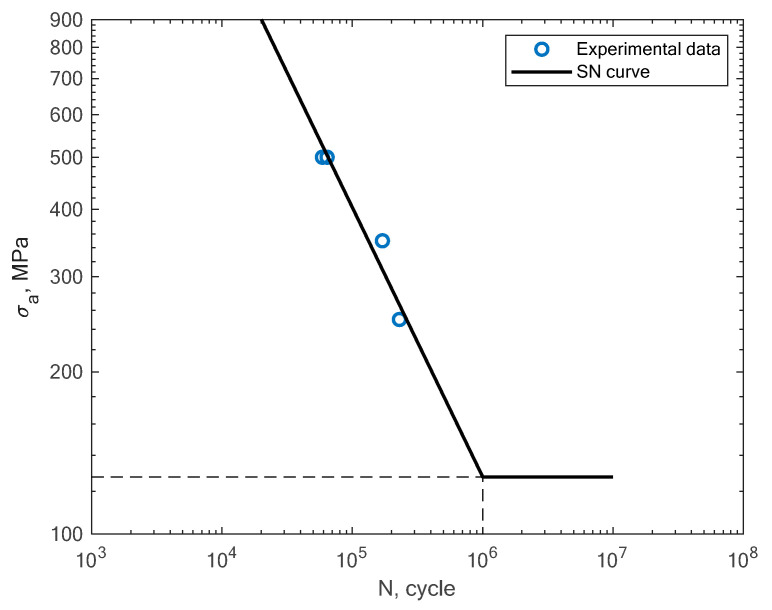
S–N curve for the experimental tension–compression (R = 0) fatigue test results reported by Gatto et al. [5] for the case of non-zero mean stress.

**Figure 20:**
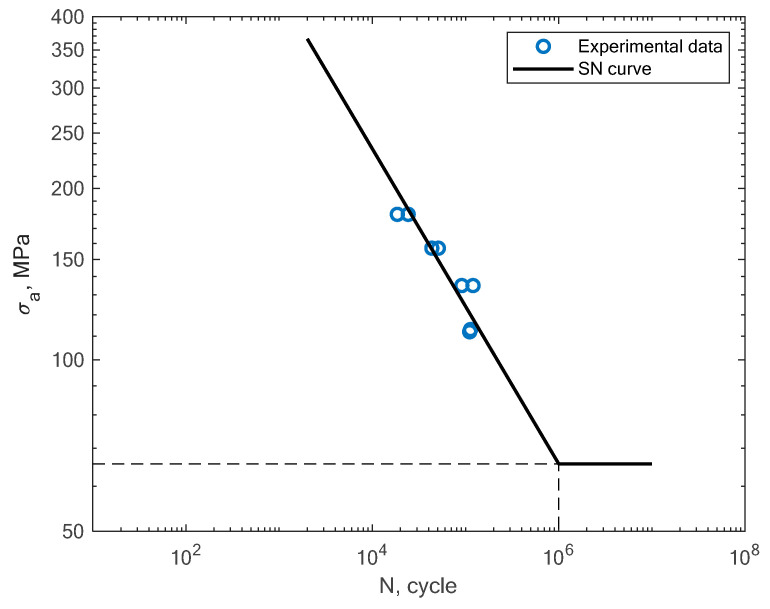
S–N curve for the experimental tension–compression (R = 0.55) fatigue test results reported by Tshabalala et al. [13] for the case of non-zero mean stress.

In Figure 11, Figure 12, Figure 13 and Figure 14, the results of the bending fatigue tests performed by Kostic et al. are presented [2]. The figures show the results for four different material conditions. The first is the condition of no heat treatment and no machining of the surface; the second is the condition of heat treatment but no machining; the third is the condition of no heat treatment and machining; and the fourth is the condition of heat treatment and machining. The experimental tension–compression S–N curves for the tests performed by Damon et al. [17] under R = −1 with two different temperature conditions, namely, room temperature (25 °C) and 400 °C, are shown in Figure 15, Figure 16, Figure 17 and Figure 18. The specimens were created with the use of the SLM technology in the horizontal and vertical directions. In Figure 19, the case of an S–N curve with non-zero mean stress under a stress ratio of R = 0 is presented. In Figure 20, the case of non-zero mean stress with a value of R = 0.55 is presented. Both these cases apply to tension–compression loading.

## 4. Discussion

**Figure 21:**
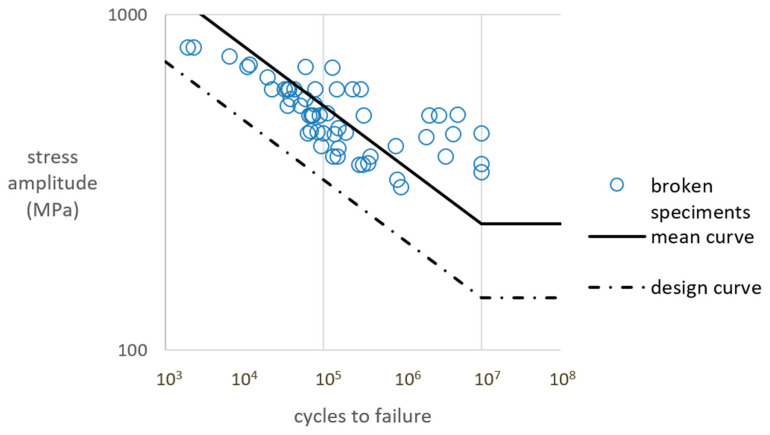
A combined reference mean and design fatigue curve on the basis of the tension–compression experimental and literature results presented in the previous section for the case of R = −1 with no high temperature effects.

In terms of practical use and the determination of parameters that might be used by engineers and designers, we can notice that there are some specific types of S–N curves that might be applicable during their praxis. Most of them incorporate simple constant amplitude tension–compression or bending states. These types of S–N curves should be divided in terms of heat treatment, loading type, specimen smoothness and stress concentrators. From the variety of S–N curves that can be found in the literature, some specific discussion points may arise. We can notice that simple fatigue tests performed under rotating bending or pure bending allow the reader to gain more information about the behavior of printed maraging steel. However, due to the fact that these results are very rare, it is currently difficult to create a reference fatigue curve for a large amount of data. On the other hand, it is possible to form such a curve for tension–compression data. On the basis of this research, including the authors’ own experimental data and the literature data, we can try to create a reference fatigue curve for the stress asymmetry ratio R = −1 with no temperature effect. This type of curve can be used in the FEM calculation procedure in order to simulate simple as well as more sophisticated loading states. Such a reference curve is presented in Figure 21. The curve was calculated on the basis of the 55 experimental points taken from our own results and the literature results. For the bending or other specific loading cases, we can notice that the results may still be insufficient to present a reference fatigue curve, which would be based on the data from only one or two papers. What can be noticed while comparing the stress amplitudes in terms of the loading state is that the bending and other states have lower curves than the tension–compression S–N curves in terms of the stress amplitude values. Table 3 presents the fatigue curve values obtained in the experimental and literature results. It also presents the reference tension–compression fatigue curve data.

As an addition to the mean curve based on the above data, there is a calculated design S–N curve with a certainty of survival equal to 97.7%. A lognormal distribution and therefore a relationship between the standard deviation and probability are assumed. The design fatigue curve is shifted by two standard deviations below the generic mean curve. This is carried out using a similar method to that of the British Standard [22].

## 5. Conclusions and Observations

On the basis of the obtained results, we can formulate the following conclusions and observations:

- The literature review has shown that there is a deficit in terms of design S–N curves for 1.2709 steel in 3D-printed form for both the tension–compression and bending loading states.
- On the basis of 55 experimental data points taken from the literature as well as our own experiments, general mean and design S–N curves for the state of tension–compression are presented.
- The presented reference mean and reference design fatigue curves, which were calculated according to the BS 7608:1993 standard for the tension–compression loading state under R = −1, may be used by engineers in their fatigue estimation of 1.2709 steel printed elements.
- The design curve has a certainty of survival equal to 97.7%.
- On the basis of the literature results, it can be noticed that the fatigue curves for the bending loading conditions are lower in comparison to the tension–compression loading state in terms of the stress amplitude values, which may be an effect of the build orientation.
- It can be noticed that the printing orientation together with the surface quality and heat treatment may influence the fatigue life of maraging steels.
- This paper serves as a reference to analyze the current state of fatigue conditions that would affect the fatigue life of 1.2709 steel used in the process of 3D printing with the use of the SLM technology.
- The next stage of this project is to present the experimental results of 3D-printed specimens with and without special surface layers that influence the durability of the material, and the authors will focus on the bending loading state in order to extend the fatigue knowledge of this material.

## Figures and Tables

**Table 1:** Important mechanical properties of 1.2709 tool steel.

*R_m_* MPa	*R*_0.2_ MPa	*E* GPa	*υ*	
1000–1200	900–1100	160–200	0.3	As-built
1670–2230	1800–2000	160–200	0.3	After age hardening
1000–1200	800–920	160–200		After solution annealing

**Table 2:** Fatigue test results under tension–compression loading and R = −1.

Specimen	*σ_a_*, MPa	*N_f_*, Cycles
MS1_AN_PLATF_5_Pr001	407	93,149
MS1_AN_PLATF_5_Pr002	407	832,264
MS1_AN_PLATF_5_Pr003	433	2,000,000
MS1_AN_PLATF_5_Pr004	459	153,479
MS1_AN_PLATF_5_Pr005	510	111,884
MS1_AN_PLATF_5_Pr006	535	50,761
MS1_AN_PLATF_5_Pr007	535	35,282
MS1_AN_PLATF_5_Pr008	446	192,177
MS1_AN_PLATF_5_Pr009	560	59,186
MS1_AN_PLATF_5_Pr010	700	10,921
MS1_AN_PLATF_5_Pr011	800	2269
MS1_AN_PLATF_5_Pr012	800	1939
MS1_AN_PLATF_5_Pr013	650	19,705
MS1_AN_PLATF_5_Pr014	750	6555
MS1_AN_PLATF_5_Pr015	600	22,496

**Table 3:** Fatigue test results under tension–compression loading and R = −1.

Reference	B	m
Experimental results	4.0531 × 10^25^	7.6277
Branco et al. [14]	1.6762 × 10^19^	5.3522
Crocollo et al. [16]	9.0667 × 10^23^	6.1542
Cruces et al. [7]	1.8687 × 10^15^	5.9366
Kostic et al. [2], no heat treatment and no machining	2.7356 × 10^9^	1.7701
Kostic et al. [2], heat treatment and no machining	7.9276 × 10^18^	5.3371
Kostic et al. [2], no heat treatment and machining	2.0597 × 10^51^	16.7676
Kostic et al. [2], heat treatment and machining	2.8920 × 10^20^	5.0046
Damon et al. [17], room temperature and horizontal direction of the printing orientation	1.0819 × 10^23^	6.6992
Damon et al. [17], room temperature and vertical direction of the printing orientation	1.3550 × 10^34^	10.3383
Damon et al. [17], high temperature of 400 °C and horizontal direction of the printing orientation	4.5591 × 10^25^	7.5174
Damon et al. [17], high temperature of 400 °C and vertical direction of the printing orientation	2.9195 × 10^30^	8.8621
Gatto et al. [5]	1.6320 × 10^10^	2.0002
Tshabalala [13]	3.7732 × 10^12^	3.6185
Reference mean curve for tension–compression	3.2076 × 10^20^	5.6825
Reference design curve with 97.7% certainty of survival	1.7861 × 10^19^	5.6825

## Data Availability

The data presented in this study are available on request from the corresponding author.
